# The congenital sternoclavicular sinus: a single-institution retrospective study of 88 patients

**DOI:** 10.1186/s13023-021-01691-x

**Published:** 2021-01-23

**Authors:** Gang Yang, Taozhen He

**Affiliations:** Department of Pediatric Surgery, West China Hospital, Sichuan University, Chengdu, 610041 Sichuan China

**Keywords:** Branchial cleft anomaly, Dermoid fistula, Children

## Abstract

**Background:**

Sinus near the sternoclavicular joint was considered as a rare congenital neck abnormality. Though it was reported as a dermoid sinus in some literatures, the embryological origin of the sinus was unclear. This study aimed at reviewing the clinical and histological characteristics and analyzing the possible embryological origin of this malformation in children.

**Methods:**

The medical records of all patients with congenital sternoclavicular sinus who underwent surgical resection between March 2018 through June 2020 were reviewed retrospectively. The clinical presentations, complications, histological examination, and treatment were analyzed.

**Results:**

Of the 88 patients with congenital sternoclavicular sinus included, the mean age of surgery was 2.73 ± 1.71 years old. The sinuses occurred on the left side in 73 (83.0%) cases. Sixty-three patients experienced sinus infection and 44 patients underwent incision and drainage before excision. All patients received surgical resection with one patient who recurred after surgery. Histopathological examination showed that the sinuses were lined by squamous epithelium in most patients. However, ciliated epithelium was observed in one patient and salivary glands were detected in two patients.

**Conclusions:**

The congenital sternoclavicular sinus should be excised promptly to prevent recurrent infection. According to the ciliated epithelium and salivary gland were found in the wall of sinus, it should be viewed as the skin side remnant of the fourth branchial cleft rather than a dermoid cyst/sinus.

## Background

Congenital cysts or sinuses resulting from embryonic structures that failed to mature or persisted in the neck are common in children. These malformations include thyroglossal duct cysts, preauricular sinuses, branchial cleft anomalies, dermoid cysts, and median cervical clefts. Sinus near the sternoclavicular joint is considered as a rare congenital neck abnormality with only about sixty cases reported in literatures [1–3]. The sternoclavicular sinus was termed dermoid sinus for its similar characteristics with dermoid cyst [4]. However, controversy on the embryological origin was raised recently. Some argued that it was more likely a congenital skin-side remnant of the fourth branchial cleft [5]. The aim of this study was to review the patients with congenital sternoclavicular sinus in the past two years retrospectively in order to analyze the clinical and histological characteristics.

## Methods

### Study design and participants

A retrospective chart review was performed to evaluate the patients with congenital sternoclavicular sinus in the Department of Pediatric Surgery, West China Hospital, Sichuan University, from March 2018 to June 2020. The study was approved by the Institutional Review Board of our hospital. All subjects participating in the study gave written informed consent. Inclusion criteria included: (1) patients younger than 14 years of age; (2) congenital sinus located near the sternoclavicular joint; (3) surgical excision performed in our hospital. The medical record, ultrasound examination and operation note were reviewed carefully to exclude other cervical fistula or cyst, such as the second brachial fistula anomaly or midline dermoid cyst. We also excluded the patients with obscure description of the precise location of the sinus in the records. Data were collected including age, sex, side of the sinus, symptoms, history of infection, prior incision and drainage (I&D), date of procedure, recurrence and histopathological finding.

### Statistical analysis

All statistical analyses were performed with SPSS software (Statistical Package for the Social Sciences; IBM Corp., Armonk, NY). The categorical variables were summarized with frequency counts and percentages. Continuous variables were summarized with means, standard deviations, median, interquartile ranges and ranges.

## Results

### Demographic features

**Fig. 1:**
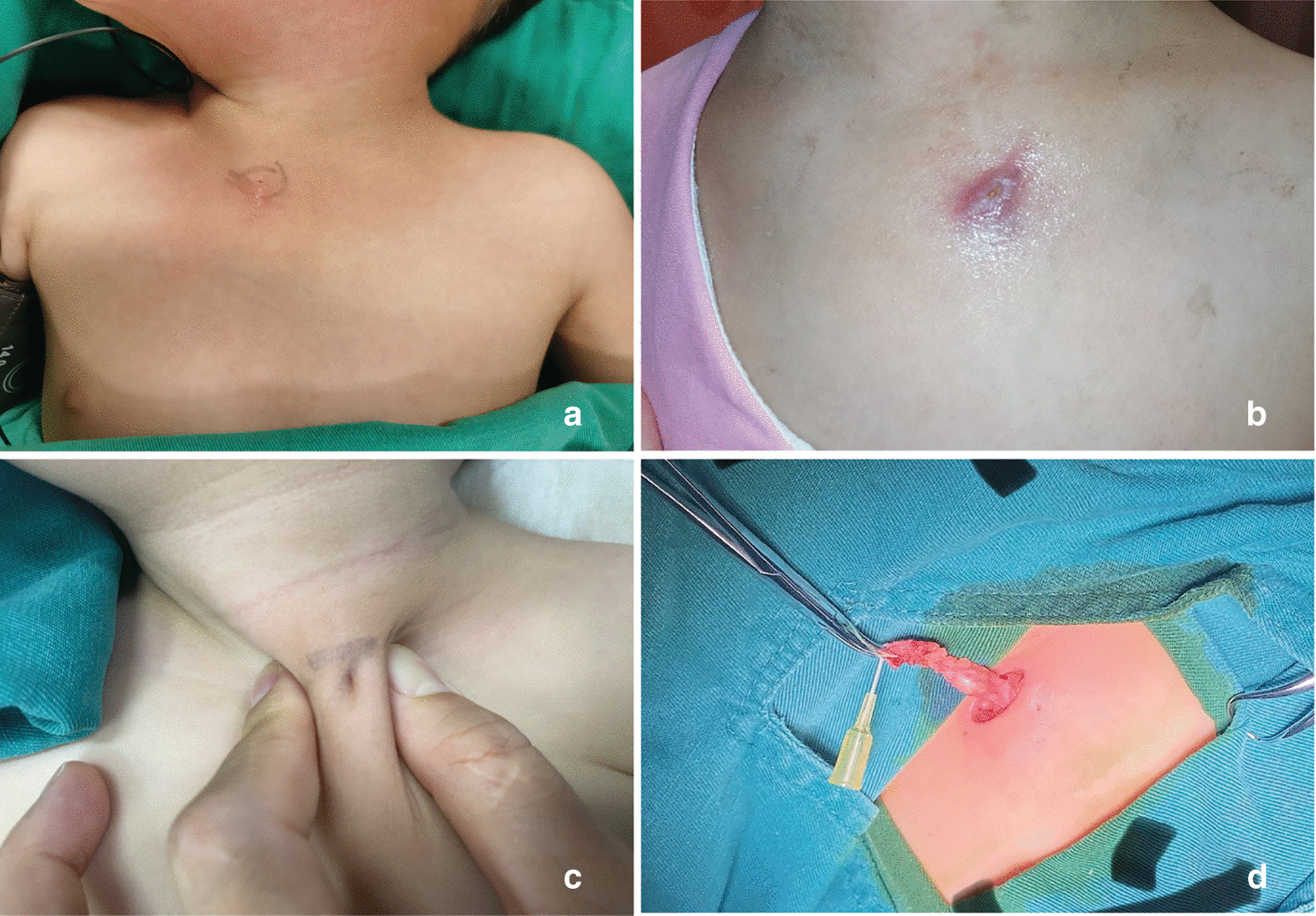
**a** Right congenital sternoclavicular sinus in a 20 months old girl. **b** Left sternoclavicular sinus in a 5 years old girl with recurrent infection. **c** Left sternoclavicular sinus in a 2 years old boy; **d** Dissection of the sinus with a catheter indwelling

Eighty-eight patients were included in the analysis. Forty-eight were girls (54.5%) and 40 were boys (45.5%). The mean age of operation was 2.73 ± 1.71 years old. The sinuses occurred on the left side in 73 (83.0%) cases, the right side in 11 (12.5%) cases and were bilateral in 4 (4.5%) cases (Fig. 1a–c, Table 1).

**Table 1:** Demographic and clinical feature of patients

Variable	No. (%)
*Sex*	
Male	40 (45.5%)
Female	48 (54.5%)
*Side of sinus*	
Left	73 (83.0%)
Right	11 (12.5%)
Bilateral	4 (4.5%)
History of infection	63 (71.6%)
Age of infection, mean (range) (year)	2.16 ± 1.55
I&D^a^	44 (69.8%)
Age of operation, mean (range) (year)	2.73 ± 1.71

*I&D* incision and drainage

^a^In patient with infection

### Clinical characteristics

All patients presented with a pit of the skin over the sternoclavicular joint along the line of the anterior margin of sternomastoid muscle since birth. In thirty-nine patients (44.3%), the parents described that there was a small amount of yellow or white solid drainage in the pit when squeezing. The drainage was mucous-like in two patients. Sixty-three patients (71.6%) experienced the history of sinus infection. The mean age of infection was 2.16 ± 1.55 years old. Of the patients with sinus infection, 44 patients (69.8%) underwent I&D for the abscess formation.

### Treatment and pathological characteristic

Excision of the sinus was performed in all patients under general anesthesia. The end of the sinus was blind at the fascia layer of the pectoralis major muscle. We did not identify any patient with the sinus penetrating the deep fascia or sternoclavicular joint capsule. The mean length of the sinuses was 11.36 ± 3.17 mm (Fig. 1d). If it was difficult to identify the sinus clearly in patient because of recurrent infection and I&D, we performed the en bloc excision of the involved skin and subcutaneous tissue including the underneath deep fascia. Recurrence occurred in one patient and underwent reoperation. Six patients had scar hypertrophy after surgery and were treated conservatively.

**Fig. 2:**
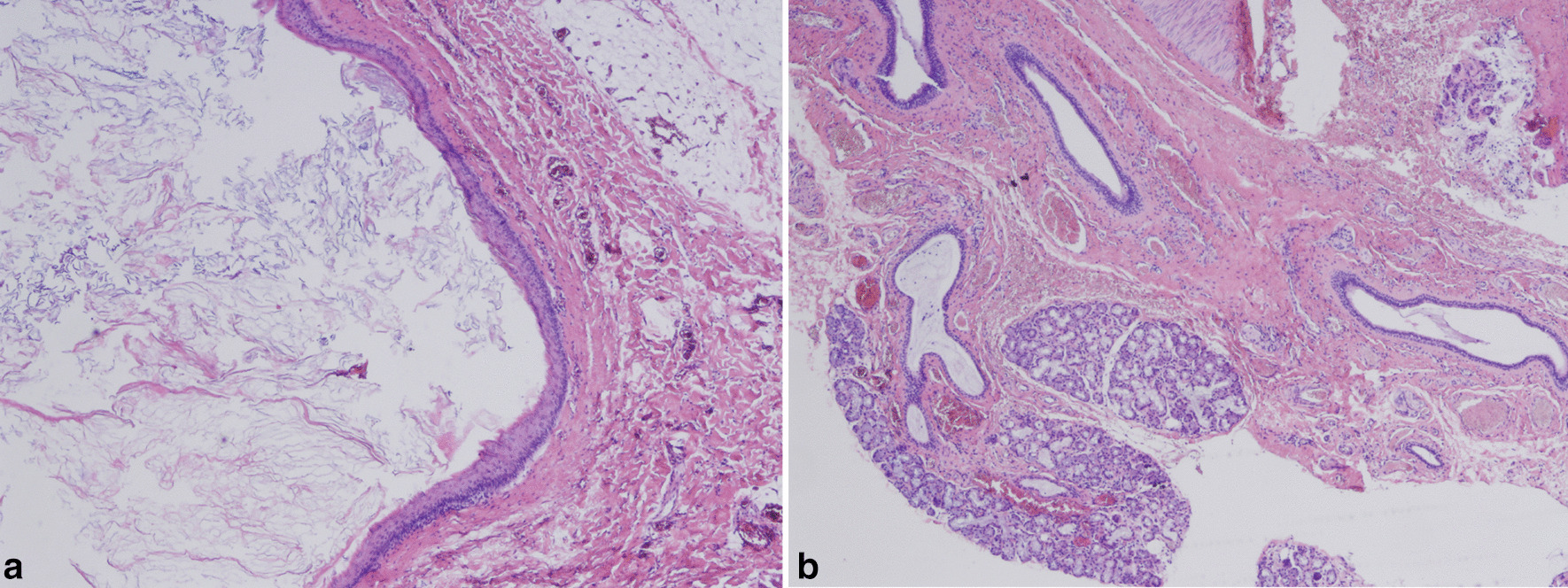
Histological examination of the fistula (×10, hematoxylin and eosin staining). **a** The sinus was lined with squamous epithelium and contained abundant keratinous material and sebum in the lumen. **b** Remnant of salivary gland was detected in the sinus

Histopathological examination showed that the sinuses were lined with squamous epithelium and contained abundant keratinous material in the lumens (Fig. 2a). However, ciliated epithelium was observed in one patient and salivary glands were detected in two patients (Fig. 2b).

## Discussion

The incidence of sternoclavicular sinus may vary around the world. This malformation was described for the first time in 1994 in 18 cases of Japanese patients [3]. Since then, a few of cases were reported in literatures [4–12]. In our study, we collected 88 patients in two years. It was more common than the second branchial anomaly and pyriform sinus in our hospital. The large number of the cases may be explained by two reasons. First, our institution is the largest tertiary hospital in west of China, which covers four provinces and more than 171 million of population. Second, though the actual incidence was unclear, most of the published reports were from Japan and China. The genetic factors may play an important role in the pathogenesis of this disease.

The embryological origin of the sternoclavicular sinus was unclear. Thus, different names have been used to describe it in literatures. In early published study, it was suggested that the sternoclavicular sinus was derived from ectoderm and designated as a dermoid fistula [9]. The other similar names included congenital sternoclavicular dermoid sinus and congenital dermoid fistulas of the anterior chest region [1]. Dermoid cysts result from entrapment of epithelial elements in the deeper tissues along embryonic lines of fusion (inclusion type of dermoid) [13]. The sternum arises from two bands of somatopleural mesenchyme near the median plane of the thorax about 6 weeks after fertilization, when the blastemal clavicles are appearing [14]. The proximal portion of the clavicle fuses to the upper part of the sternum. Histologically, dermoid cyst/sinus is lined by squamous epithelium with adnexal structures of ectodermal origin (hair follicles, sebaceous glands and sweat glands) [15]. These histopathological features were observed in most sternoclavicular sinuses in our series.

However, there were two characteristics of the sternoclavicular sinus that cannot be explained by the hypothesis of dermoid sinus. Firstly, in accordance with previous reports, most of the sinuses are in the left side [2, 3, 6]. The frequently involved area of dermoid cyst is the periorbital area, followed by the nasal dorsum, submental area, and the suprasternal area [16]. The nasal, submental and suprasternal dermoid are in the midline. As to the periorbital dermoid, we did not find it to occur in one side more likely than the other side [17]. Another similar malformation is preauricular sinuses [18]. They are not of true branchial cleft origin and related to the embryonic ectodermal mounds (auditory hillocks) that essentially form the auricles of the ear. The preauricular sinuses also have race/ethnicity difference in the occurrence and are more commonly on the right side. But, the lining cells of preauricular sinuses are stratified squamous epithelium. The ciliated columnar epithelium or salivary gland are never seen in it. The second characteristic of sternoclavicular sinus not conforming to dermoid sinus was ciliated columnar epithelium or salivary gland being detected histologically in the specimen. The salivary gland and ciliated epithelium are endodermal origin and they can present in the branchial remnant fistula [19].

All these findings suggested that the congenital sternoclavicular sinus was related to the fourth branchial remnant fistula. Both third and fourth branchial anomalies are rare congenital malformation of the neck. These fistulas enter the pyriform sinus and their clinical differentiation is difficult [20]. The fourth arch anomaly usually presents as cyst in newborn and suppurative thyroiditis outside the neonatal period [21]. Most of the symptomatic pyriform sinuses (more than 90%) are located on the left side [22]. Theoretically, the entire course of the fourth branchial fistula originates at the apex of the pyriform sinus, descends beneath the aortic arch, and then ascends anterior to the carotid artery to end in the vestigial cervical sinus of His [23]. Actually, the fistula of complete remnant of the fourth branchial apparatus has never been reported. The external opening site of the fourth branchial fistula is near the sternoclavicular joint area. Therefore, the sternoclavicular sinus may be the skin side of the fourth branchial remnant fistula. This can also explain the findings of ciliated epithelium and salivary gland in the histological examination of the sinuses. The hypothesis was also supported by study performed by Ohno et al. [5]. In the study, the authors described the presence of ciliated columnar epithelium and salivary gland in congenital cutaneous fistula situated near the sternoclavicular joint.

The main complication of the sternoclavicular sinus was infection and abscess formation. The infection can occur as early as 2 months old. I&D should be performed in most patients presenting an abscess formation. Moreover, the infection tended to recur in a short period even after I&D. Surgical resection of the sinus was indicated in all patients diagnosed with sternoclavicular sinus. Complete resection was simple in patient without previous infection for the short course of sinus. However, it would be difficult to identify the sinus in patient with recurrent infection and prior I&D. The en bloc excision of the involved tissue was sufficient to prevent the recurrence.

The observational and retrospective nature of this study was an important limitation. As an academic institution and tertiary medical center, there may be more patients with rare diseases referred to our hospital. The prevalence may be overestimated.

## Conclusion

In this study, we presented the largest single-center congenital sternoclavicular sinus series. According to the location of sinus, tending to occur in left side, and ciliated epithelium or salivary gland detected in the sinus, we consider that the sinus was the skin side remnant of the fourth branchial cleft rather than a dermoid cyst/sinus.

## Data Availability

The datasets used and/or analyzed during the current study are available from the corresponding author on reasonable request.

## Abbreviation

I&D: Incision and drainage
